# Supplementary material for: Coordinated adaptations define the ontogenetic shift from worm- to fish-hunting in a venomous cone snail
Source: Nat Commun. 2023 Jun 13;14:3287. doi: 10.1038/s41467-023-38924-5 (PMC10264353; doi:10.1038/s41467-023-38924-5)
Supplement: Supplementary file 1 — Supplementary Information [file 41467_2023_38924_MOESM1_ESM.pdf]

Coordinated adaptations define the ontogenetic shift from worm- to fish-hunting in a venomous cone snail

Supplementary Figure 1: Morphogenesis of the venom apparatus in *C. magus*.

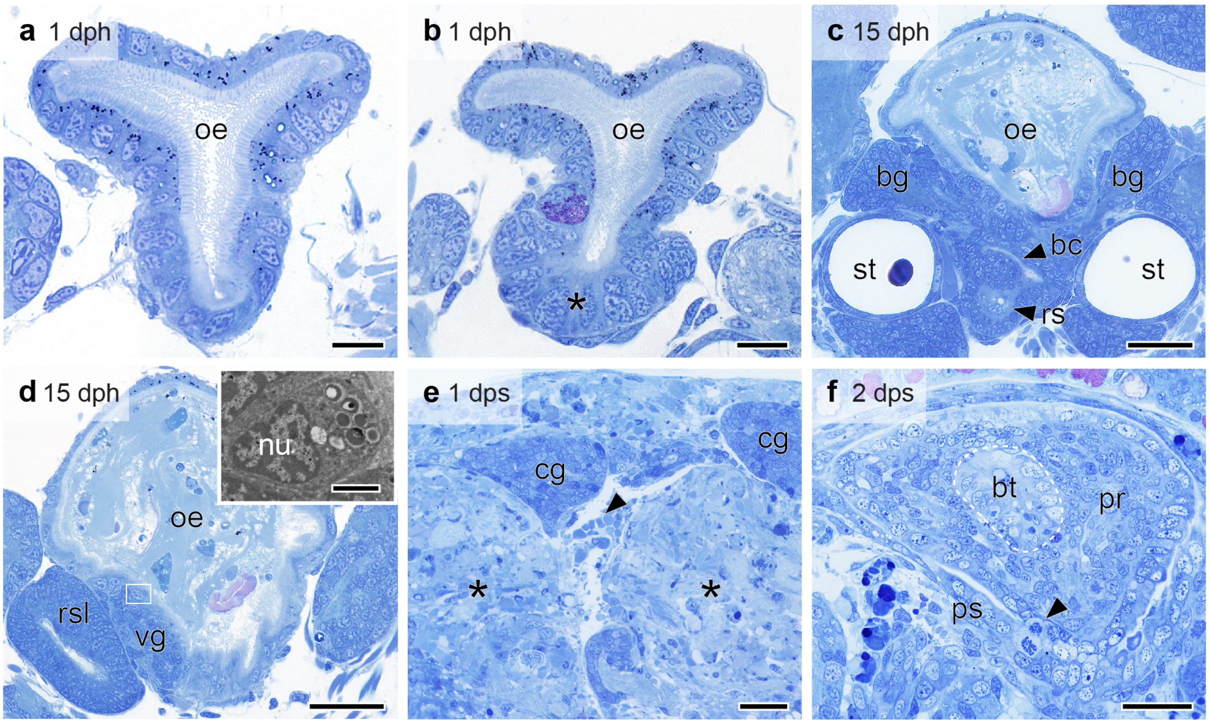

Elements of the venom apparatus were initiated from the larval foregut. (**a**) At 1 day post-hatching (1 dph), the oesophagus (oe) consisted of a simple tube of ciliated epithelium connecting the mouth to the stomach, but (**b**) ~ 50 µm in from the mouth, cross-sections revealed a patch of enlarged non-ciliated cells (asterisk) embedded in the ventral wall of the oesophagus that differentiated into the buccal cavity and radular sac. Scale bars (**a**,**b**) = 10 µm. Two specimens were sectioned with similar results. (**c**) In late larva (15 dph), the radular sac (rs) had formed as an out-pocketing of the buccal cavity (bc) and both structures could be seen as interconnected chambers beneath the oesophagus, at the level of the statocysts (st). Scale bar = 30 µm (**d**) Immediately posterior to the buccal commissure, the prospective venom gland (vg) was evident as an accumulation of secretory cells embedded in the ventral wall of the oesophagus, adjacent to the long arm of the radular sac (rsl). The cytoplasm of the venom gland cells was filled with spherical secretory granules (~ 700 nm in diameter), accumulating at the apical region (inset, transmission electron microscopy). Scale bars: (**d**) = 30 µm, inset = 2 µm. (**e**) 24 h after the onset of metamorphosis (1 day post-settlement, dps), the two masses of involuted velar cells arising from the resorption of the velum (asterisks) could be seen on each side of the head, beneath the cerebral ganglia (cg). Black arrowhead indicates the position of the future buccal tube. Scale bar = 30 µm. (**f**) 48 hours after the onset of metamorphosis (2 dps), the proboscis (pr) and its sheath (ps) had formed around the buccal tube (bt, white dotted line). Black arrowhead indicates mitotic profile of dividing cell. Scale bar = 30 µm. Other abbreviations: bg, buccal ganglia; nu, nucleus. Four specimens at 15 dph, 1 dps and 2 dps were sectioned with similar results.

Supplementary Figure 2: Anatomy of the juvenile radular tooth and venom gland.

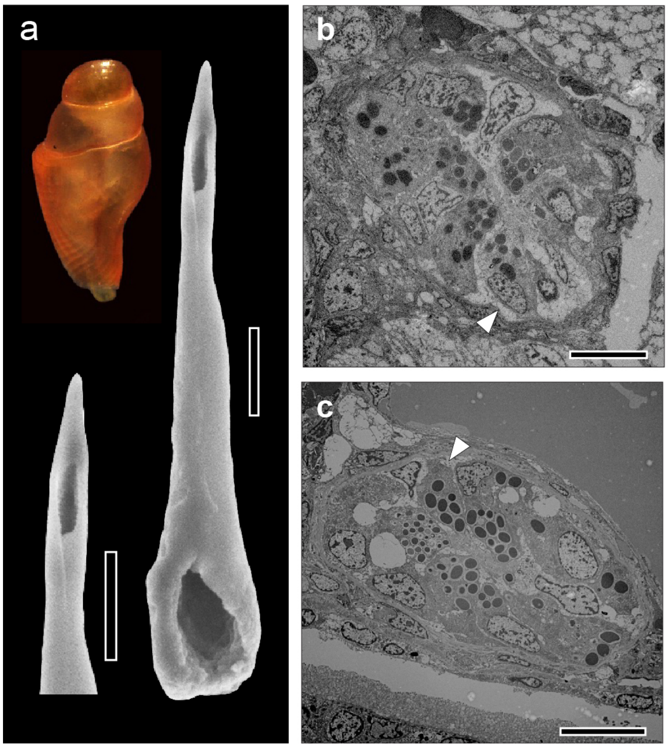

Proximal venom gland	
**Diameter**	42.6 µm
**Cell type**	Cuboidal
**Secretion**	Holocrine
**Granule size**	1.9 µm
**Lumen**	Constricted

Distal venom gland	
**Diameter**	41.6 µm
**Cell type**	Cuboidal
**Secretion**	Holocrine
**Granule size**	1.7 µm
**Lumen**	Constricted

Vermivory in juvenile *C. magus* was facilitated by short, unbarbed radular teeth. (**a**) Scanning electron micrograph of the juvenile radular tooth. The juvenile radular tooth (6 dps) was short in absolute and relative length, with a wide base and a large opening. It lacked apical barbs, blades and serrations. Shell length = 1.6 mm. Scale bars = 10 µm. (**b**,**c**) Transmission electron microscopy revealed the juvenile VG was structurally homogenous along its length. In both proximal and distal regions, epithelial cells lining the VG were filled with secretory granules, and the lumen was highly constricted. The dissociation of the secretory cells from the basal lamina (white arrowheads) and rupture of their membrane indicate that granules are released through a holocrine process. Scale bars = 10 µm. Five radular teeth were dissected and measured (see **Supplementary Data 1**) and four juveniles at 6 dps were sectioned with one selected for TEM.

Supplementary Figure 3: Radular tooth morphometry in Conidae.

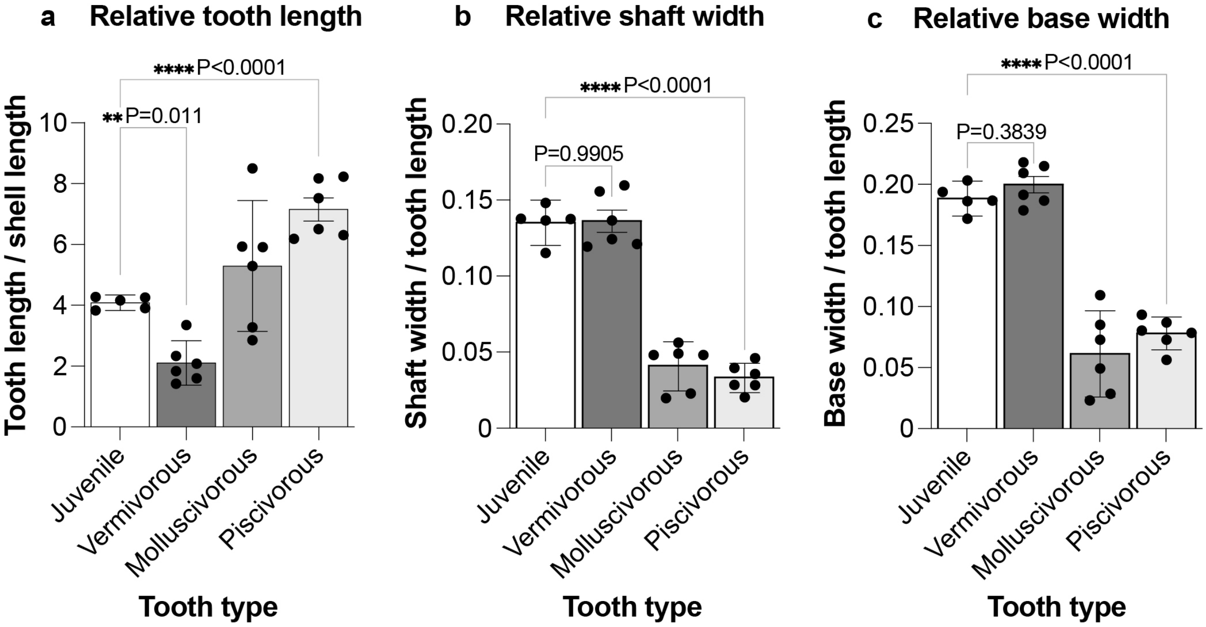

Morphometric analysis of radular teeth reveal vermivorous-type tooth of juvenile *C. magus*. (**a**) The relative length of the tooth (T_L) can be expressed as the percentage of the shell length (S_L) with the ratio $T_L / S_L \times 100$. A short tooth is the ancestral state and is found in vermivorous Conidae and juvenile *C. magus*. A longer tooth is a derived condition and is characteristic of piscivorous and molluscivorous species. (**b**,**c**) The stoutness of the tooth can be measured by the relative width of the shaft and base. A thicker tooth with a wide base is the ancestral type. The tooth of recently evolved piscivorous and molluscivorous species is thin and the base poorly defined. (**b**) The relative width of the shaft is measured by the ratio S_W / T_L, where S_W is the maximum shaft width. (**c**) The relative width of the base is measured by the ratio B_W / T_L, where B_W is the maximum base width. Juvenile tooth: N = 5. Vermivorous type: *C. naranjus*, *C. flavusalbus*, *C. ventricosus*, *C. miliaris*, *C. borgesi*, *C. abbreviatus*. Molluscivorous type: *C. amadis*, *C. episcopatus*, *C. pennaceus*, *C. ammiralis*, *C. marmoreus*, *C. textile*. Piscivorous type: *C. timorensis*, *C. achatinus*, *C. ermineus*, *C. striatus*, *C. leebmani*, *C. catus*. Data for adult radular teeth are from Mosquera and Massilia, 2002. Statistical significance was defined as $P < 0.05$ using a one-way ANOVA test and data are presented as means ± SEM. Source data are provided as **Supplementary Data 1**.

Supplementary Figure 4: Anatomy of the adult radular tooth and venom gland.

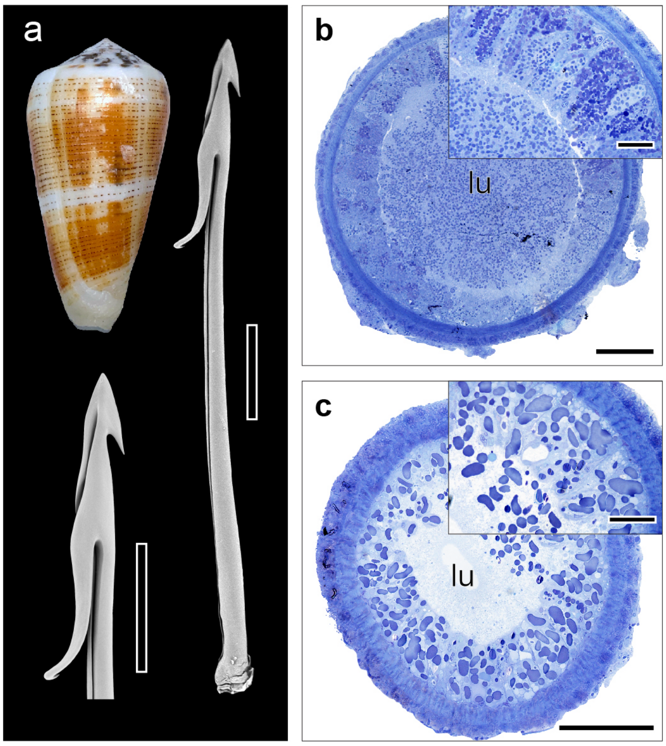

Proximal venom gland	
**Diameter**	580.7 µm
**Cell type**	Columnar
**Secretion**	Holocrine
**Granule size**	5.4 µm
**Lumen**	Filled with granules

Distal venom gland	
**Diameter**	359.8 µm
**Cell type**	Columnar
**Secretion**	Holocrine
**Granule size**	25.1 µm
**Lumen**	Cell debris

Piscivory in adult *C. magus* was facilitated by long, harpoon-like radular teeth. (**a**) Scanning electron micrograph of the adult radular tooth. The adult radular tooth was long and narrow, and the base poorly defined. It had a barb and blade at the apex and the long, hooked accessory process used to tether fish prey. The basal ligament was no longer visible after processing the sample. Shell length = 53 mm. Scale bars = 500 µm. (**b**,**c**) Histology combined with light microscopy revealed that the proximal and distal regions of the adult venom gland (VG) differed in their ultrastructure and secretory products. (**b**) The lumen of the proximal VG was filled with small granules at different stages of maturity. Scale bars: (**b**) = 100, inset = 30 µm. (**c**) In contrast, cellular debris and a few granules were found in the lumen of the distal VG. The secretory cells in this region of the VG were filled with large granules, secreted in the lumen through holocrine process. Scale bars: (**c**) = 100, inset = 30 µm. Five radular teeth were dissected and a single VG sectioned from the laying female.

Supplementary Figure 5: Somatostatin-like conotoxins from *C. magus*.

a

```
C. magus       Consomatin_M2  NGSGSSNQKEAQICIWKVCPPTPW
consomatins    Consomatin_M3  LRSSDSSVRDAQPCIWKICPPSPW
Annelid        C. teleta      -----QASSGAQICIWKVCPPTPW
SSRP-like      P. elegans     -----QSSSGAQICIWKVCPPSPW
               L. satsuma     -----QSRSGAQICIWKVCPPSPW
               P. dumerilii   ----QQPSSGSKICIWKVCPPSPW
                                           **** *** **
```

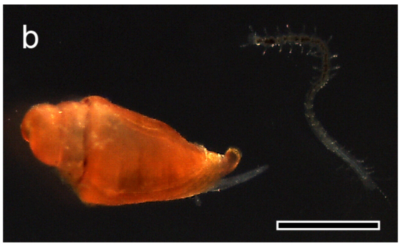

Somatostatin-like conotoxins (consomatins) from *C. magus* share strong similarity with annelid somatostatin-related peptides-like (SSRP-like). (**a**) Multiple sequence alignment of consomatins from *C. magus* with signalling SSRP-like peptides from diverse annelid species. Both *C. magus* consomatins were found in the juvenile and adult transcriptomes, although upregulated in the juveniles. Annelid sequences are from Koch et al, 2022. (**b**) The upregulation of consomatins showing similarity with annelid SSRP-like peptides in the juveniles was consistent with their vermivorous diet. Juvenile *C. magus* (10 dps) preying on a syllid worm, showing the proboscis extended. Scale bar = 1 mm.

Supplementary Figure 6: Sequence alignments of juvenile dominant transcripts.

O1 superfamily

```
                      ----------Signal--------- ----------Propeptide------- -----------Mature-----------
O1_M6.53(42%)         MKLTCVLIVAVLFLTACQLITADDSRDEYRAVRSSDEVQNSKSTRACSESGDGCLTRNCCPGLTCSGPQQGPVCH   75
O1_M6.54(7.6%)        MKLTCVLIVAVLFLTACQLITADDSRDEYRAVRSSDEVQNSKSTRACSESGDGCRTRSCCPGLRCSGTHQARVCH   75
C.ventricosus_Q9BP94  MKLTCVLIIAVLFLTACQLTTA-ETRDEYRAVRSSDEVRNSRSTRDCSGSGYGCKNTPCCDGLTCRGPHQGPIC-   73
                      ******** ********** **   ************* ** *** ** ** **    ** ** * *  *   *
```

L superfamily

```
L_M14.14i(11.8%)      MKLSVTFIVVLMLTTSLTCGYSLFSNNGER-AFGPHDPDTAN--HREERASRECNPPCTGLAMCQGGKCGYKRFG   72
L_M14.14ii(7.7%)      MKLSVTFIVVLMLTTSLTCGYSLFPNNGER-AFGPRDPDAAN--HREERASRECNPPCTGLAMCQGGKCGYKRFG   72
C.ebraeus_UMA82769    MKLLVTFIVVLMLATSLTYGFSLFSNNGERRAFEPRDPDAADQLVREERATRTCNPPCTGLAMCQSGTCGYVRF-   74
                      *** ********* **** * *** ***** ** * *** *     ***** * ************ * *** **
```

B2 superfamily

```
B2_M1(9.7%)           MLRLIIAAAVLLSACLAYPQRREGAPADAANLQSFDPALMPMQGMQGGQMAGMA------------GGQFLPFNPNLQMGYKRDF
Turritoxin_Ubs_15     MLRLII-AAVLASACLAFPQRRDGFPGEAANLKAFGPDMQGMQAMPGGMPGGMPGAMPNMQPMQPMPGQFLPFNPNLGMGFKRAA
Turritoxin_Ccr_06     MLRLII-AAVLASACLAFPQRRDGFPGEAANLKAFGPDMQGMQAMPGGMPGGMPGAMPNMQPMQPMPGQFLPFNPNLGMGFKRAA
                      ****** **** ***** **** * *  ****  * *    ** * **   **              ********** ** **

B2_M1(9.7%)           DENLEKRKQHSQFNADENKAPFDSEENF-------MNFLHNEKGDKHPFANVDS-AATDLGQFEPSAEN-EDGKFRFFDKEQ   146
Turritoxin_Ubs_15     DENLEKRKHHSKFQ-DENKSPFDSPADSMLGNFNFGKFL-QENPDNIPFANMENANPGDLGNFEPNAEDSKEGHFRFFDQQQ   164
Turritoxin_Ccr_06     DENLEKRKHHSKFQ-DENKSPFDSPADSMLGNFNFGKFL-QENPDNIPFANMENANPGDLGNFEPNAEDSKEGHFRFFDQQQ   164
                      ******** ** *  **** ****              **  *  *  ****       *** *** **    * *****  *
```

O2 superfamily

```
O2_M6.62(3.8%)        MEKLTILLLVAAVLMSTQALNQEQRQQAKNNLLSKRKPSAERWRRDCTSWFGSCSVDSDCCSNSCDQSYCELYAFPFRAV   80
C.pennaceus_AAG60457  MEKLTILLLVAAVLMSTQAQNQEQRQQAKINFLSKRKPSAERWRRDCTSWFGRCTVNSECCSNSCDQTYCELYAFPSFGA   80
                      ******************* ********* * ******************** * * * ******** ********
```

T superfamily

```
T_M1.18(1.1%)         MLCLPIFIILLLFLSPAVSFPVESKLESLWTRMLSRDLGEEHQKNNRQSQAECCPPHIEPCTPCTIYKLNK   71
C.judaeus_DAZ86876    MLRFPIFLILLLVLSPAAGFPVESKLENLWARMLSRDFGEERHTNNRRILPLCCMQQQSPCSSCIPT----   67
                      **  *** **** ****  ******** ** ****** ***   ***     **     **  *
```

The new O1-superfamily juvenile transcripts O1_M6.53 and O1_M6.54 contributed 42.0% and 7.6% of total expression, respectively. A blastp search revealed these were most closely related to vermivorous conotoxins from *C. ventricosus*, *C. distans* or *C. flavidus*. The new L-superfamily juvenile transcripts L_M14.14i and L_M14.14ii also contributed significantly to overall expression (11.8% and 7.7%, respectively) and again shared high sequence similarity with precursors from a vermivorous species (*C. ebraeus*). The new juvenile transcript B2_M1, the third most highly expressed transcript (9.7%), was closely related to peptides isolated from the venom glands of Turridae species *Unedogemmula bisaya* and *Crassispira cerithina*[3]. The new juvenile O2-superfamily transcript O2_M6.62 (3.8%) shared similarity with γ-PnVIIA from the mollusc-hunter *C. pennaceus*, while the new T-superfamily transcript T_M1.18 shared similarity with a precursor from the vermivorous *C. judaeus*. NCBI accession numbers are indicated after the species names. Juvenile sequence names are in bold type and relative transcriptomic expression indicated in brackets. Signal, propeptide and mature regions are indicated and cysteines residues in the mature sequence are highlighted in yellow. Asterisks below sequences indicate identities.

Supplementary Figure 7: Phylogeny of the O1-conotoxin gene superfamily.

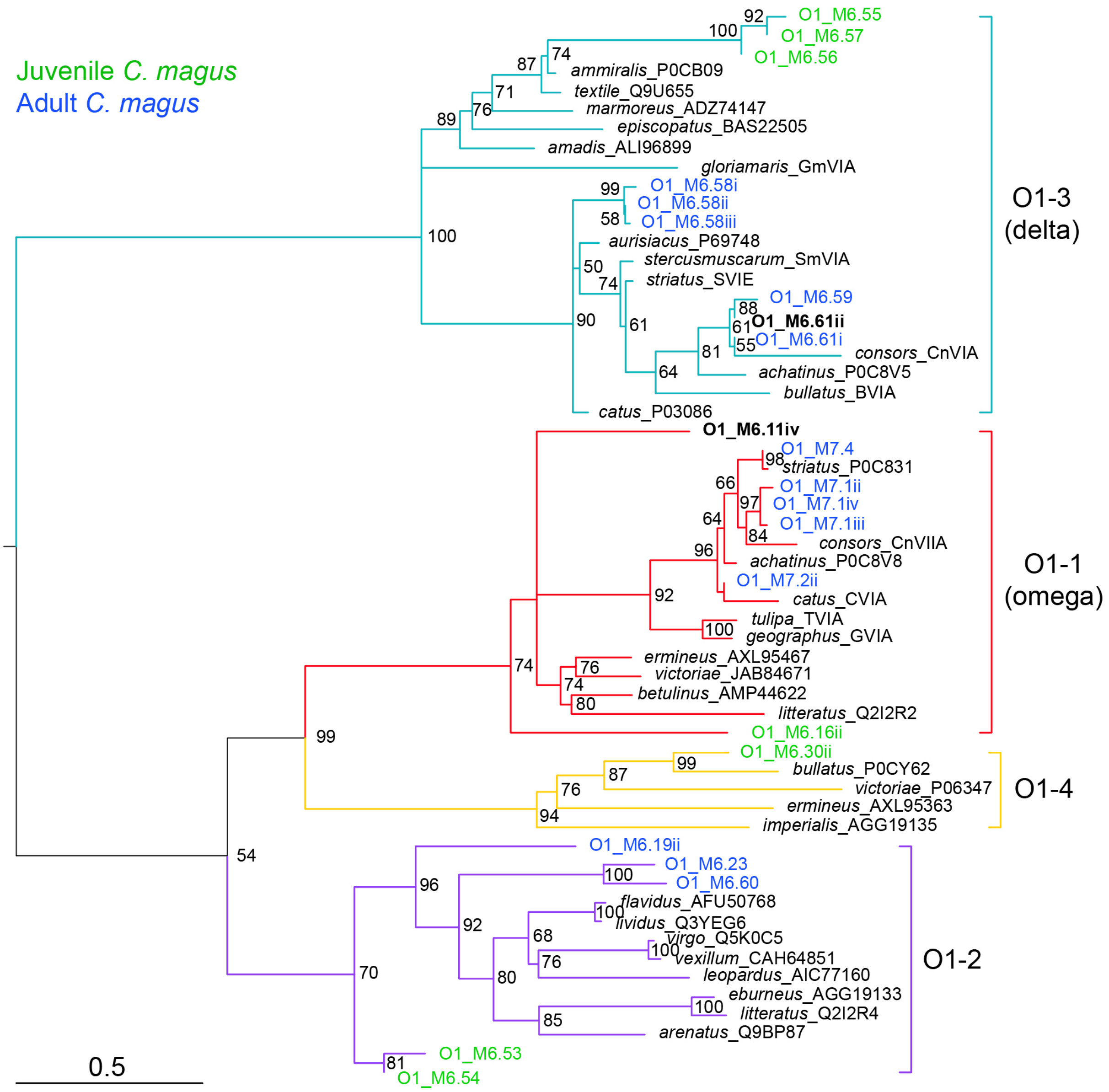

Maximum likelihood tree of the O1-conotoxin gene superfamily, based on full length precursor sequences from vermivorous, molluscivorous and piscivorous Conidae. The tree was reconstructed using the JTT+G4 model and is displayed as midpoint rooted. Paralog groups are indicated. Bootstrap support values are indicated, and branches with support < 50% are collapsed into polytomies. Scale bar indicates expected substitutions/site. GenBank accession numbers are indicated after species names. Green and blue sequence names are from juvenile and adult *C. magus*, respectively, and sequence names in bold type are shared between embryos, juveniles and adults.

Supplementary Figure 8: MALDI- and LC-MS support venom ontogeny in *C. magus* and the heterogenous distribution of conotoxins along the adult venom gland.

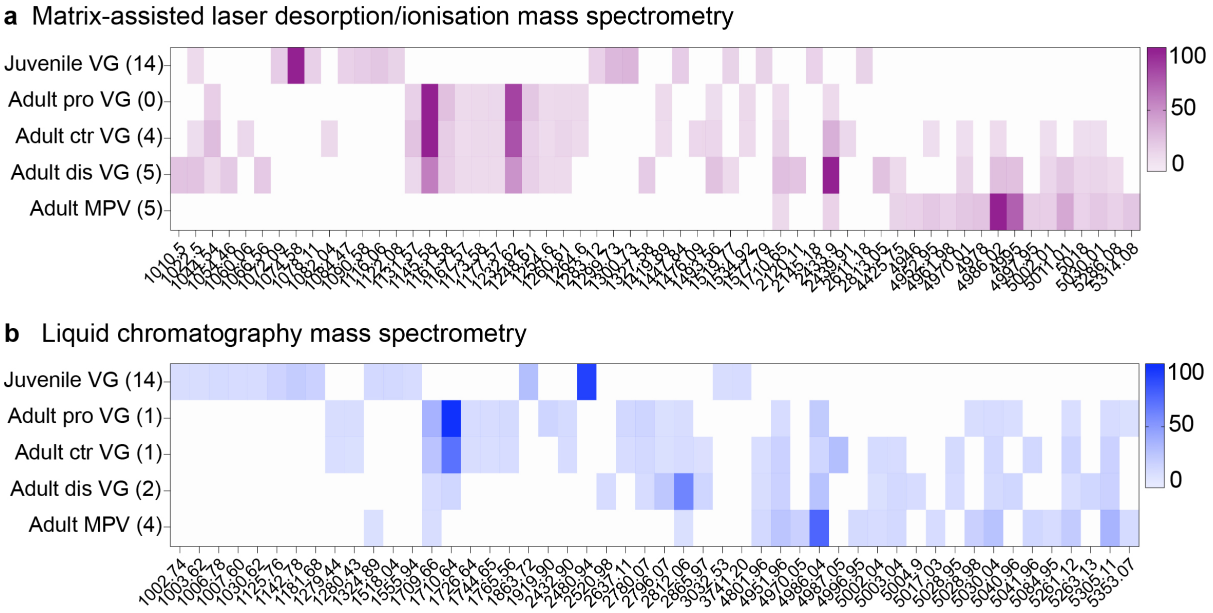

(**a**) MALDI-MS (TIMS TOF Flex MALDI-2, Bruker) revealed different suites of peptides in the juvenile and adult venom gland (VG), as well as the heterogenous distribution of conotoxins along the adult VG. Peptides in the 4–6 kDa range were restricted to the adult VG, and dominated the milked predatory venom (MPV). The heatmap was built using the 15 most intense peaks identified in each sample. Threshold for equivalent masses between samples was set at 0.01 Da. Number of peptides unique to each sample is indicated in brackets. While MALDI-MS is a useful technique for whole venom profiling, this approach suffers a number of limitations preventing the detection of the full venom complexity. Additional LC-MS data were therefore acquired. (**b**) LC-MS (ZenoTOF 7600, SCIEX) also revealed different peptide masses in the juvenile and adult VG. The heatmap was built using the 15 most intense masses identified in each sample. Masses within 0.01 Da and eluting within 0.2 min between two samples were considered equivalent. Number of peptides unique to each sample is indicated in brackets. Other abbreviations: pro, proximal; ctr, central; dis, distal. Source data are provided as **Supplementary Data 4**.

Supplementary Figure 9: Evidence of glycosylated peptides in the adult *C. magus* venom gland and milked predatory venom.

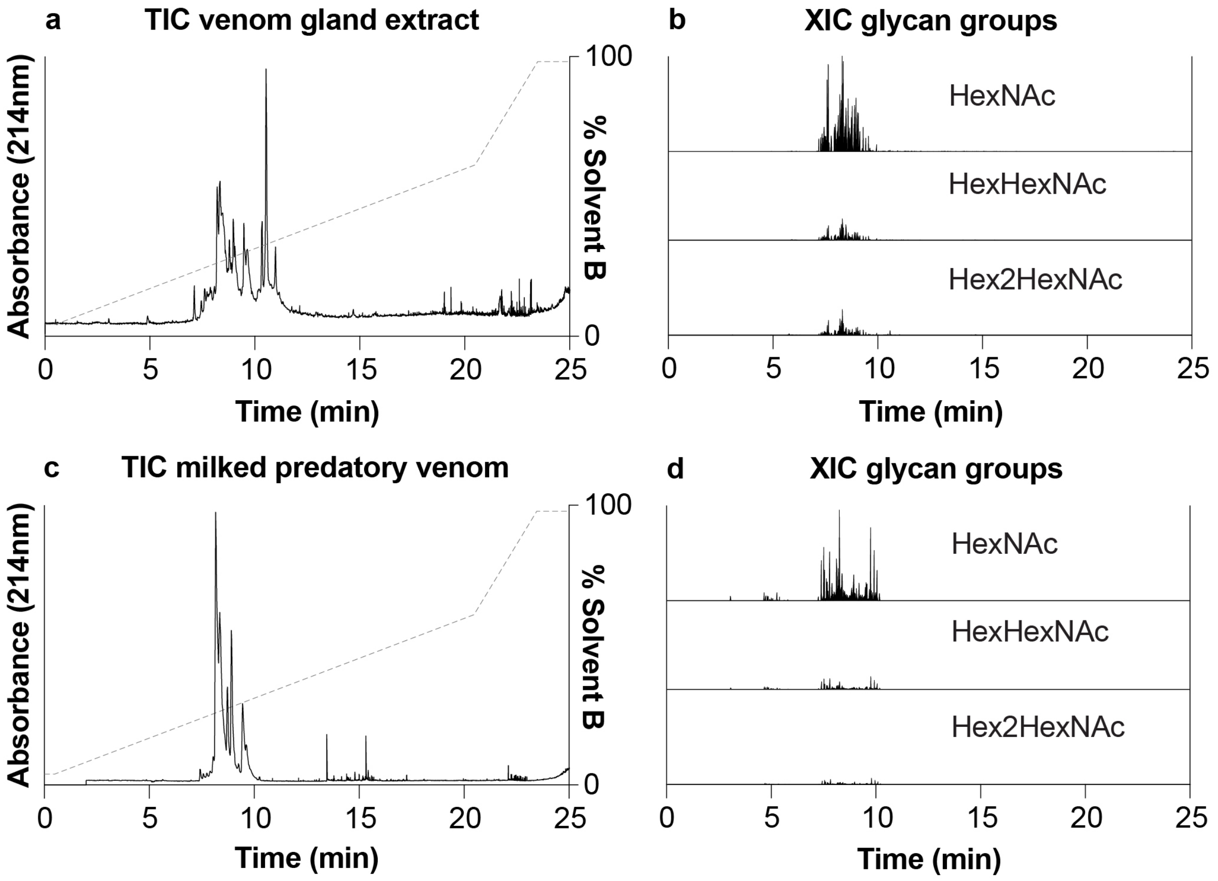

LC-MS revealed the presence of carbohydrate marker ions in the adult *C. magus* venom gland and milked predatory venom. (**a**) Total ion current (TIC) chromatogram of the adult venom gland extract and (**b**) extracted ion chromatograms (XIC) corresponding to the masses of carbohydrate markers HexNAc (m/z 204.08 Da), HexHexNAc (m/z 366.13 Da) and Hex2HexNAc (m/z 528.19 Da). (**c**) TIC chromatogram of the milked predatory venom, obtained from 10 individual injections and (**d**) extracted ion chromatograms (XIC) corresponding to the masses of HexNAc, HexHexNAc and Hex2HexNAc.

Supplementary Table 1: Identification of conotoxins in the adult *C. magus* venom gland and milked predatory venom by liquid chromatography-tandem mass spectrometry.

Conotoxin name	Mature peptide sequence	Proximal	Central	Distal	MPV
A_M1.2 (MII)	GCCSNPVCHLEHSNLC	*	*	*	
A_M1.17	NGRCCHPACGKNYSC	*	*		
A_M4.5	APELVVTATTTCCGYDPMTWCPSCMCTYSCPHQRKKP				*
A_M4.7	APELVVTATTTCCGYNPMTICPPCMCTNSCPPKRKP		*	*	
Conkunitzin_M9.10	FIYGGCGGNGNRFLTQDACLNTCIYPLTGNQGY	*	*	*	*
Conkunitzin_M9.12	FFFDITSRQCKMFTYGGCEGNGNNFNRTYDCLKMCVYSAG		*	*	*
Conkunitzin_M14.9	IYYNSARKQCLRFTYHGEGGNANNFLHTFDCQHTCLYTG	*	*	*	*
Conomarphin_M2	TRNYYVYSARPKNSWWS	*			
Conoporin_M1	> 50 a.a.	*	*	*	*
Conoporin_M6	> 50 a.a.	*	*	*	*
M_M3.4	CCGPGGSCPVYFRDNFICGCC	*	*	*	*
M_M3.7	RCCGEGSSCPKYFRNSQICHCC				*
O1_M6.15	CASYGKPCGIYNDCCNTCDPARKTCT	*	*	*	*
O1_M6.19	KSWSCVEHGDSCKTNICCAGLTCLRTHAINICLYLMPM		*		
O1_M6.60	VRASDSCAERGEPCQHRRCCPDLICVGGHVGRRCRYPYNYYYN	*	*	*	*
O1_M6.61	DECYPPGTFCGIKPGLCCSARCFSFVCISFEF			*	
O1_M7.1 (MVIIA)	CKGKGAKCSRLMYDCCTGSCRSGKC	*	*	*	*
O1_M7.2 (MVIIB)	CKGKGASCHRTSYDCCTGSCNRGKC	*	*	*	*
O1_M7.4	CKGKGAPCRKTMYDCCSGSCGRRGKC	*	*		*
S_M8.1	SLAIGCTGTCYQSSRCDGKCDCDHSKNCYCSGKGHHHPGCTCTCQM		*		*
S_M8.3	SLAIGCTGTCYQSSRCDGKCDCDHSKNCYCSGKGHHHPGCTCTCRM			*	
S_M8.6	QGCTGTCYQSSRCDGKCDCDHSKNCYCSGKGHHHPGCTCTCQM		*	*	*
T_M2	FLGAVFGGISLLSTLIDALSS	*		*	
T_M3	IFSSIADNVFKVAPALAGAIIPIIADLLNGAEVVE	*			
T_M5	IFSSMTEYVSKVIPDLADAIIPFITDLVNGAEVV	*			
T_M6	FFSSIAKLVSKVAPAVAGAIIPVITDLVNGA	*	*	*	*
T_M7	IFSSIAKLVSKVAPAVAGAIIPVITDLVNGA	*	*	*	
T_M8	FFSSIAKLVSKVAPALAGAIIPVITDLVNGA	*	*	*	
T_M9	IVSSMTEYVSKVIPDLADAIIPFITDLVNGAEVV	*			
T_M10	IVSSMTEYVSKVIPDLADAIIPVITDLVNGA	*		*	

Peptides identified by LC-MS/MS in the adult proximal, central and distal venom gland (VG) and milked predatory venom (MPV) are indicated. ProteinPilot and Mascot were used to identify sequences from MS/MS datasets. For ProteinPilot, only peptides with ≥ 2 tryptic fragments at a confidence of 99 and a false discovery rate < 1% were considered genuine. For Mascot, only peptides with ≥ 2 tryptic fragments, individual peptide scores > 60 and a significance threshold < 0.05 were selected. MS/MS spectra were searched against a database combining sequences generated from our RNA-seq experiments and all published *C. magus* conotoxins (see **Supplementary Data 2**). Red asterisks indicate sequences found in matching transcriptomes from the parent.

Supplementary References

1. Mosquera, E. R. & Massilia, G. R. Evaluation of character state polarity of *Conus* radular tooth characters. *Boll. Malacol.* **38**, 175–194 (2002).
2. Koch, T. L. et al. Reconstructing the origins of the somatostatin and allatostatin-C signaling systems using the accelerated evolution of biodiverse cone snail toxins. *Mol. Biol. Evol.* **39**, 4 (2022).
3. Gonzales, D. & Saloma, C. A bioinformatics survey for conotoxin-like sequences in three turrid snail venom duct transcriptomes. *Toxicon* **92**, 66–74 (2014).
