# Supplementary material for: Coordinated adaptations define the ontogenetic shift from worm- to fish-hunting in a venomous cone snail
Source: Nat Commun. 2023 Jun 13;14:3287. doi: 10.1038/s41467-023-38924-5 (PMC10264353; doi:10.1038/s41467-023-38924-5)
Supplement: Supplementary file 3 — Reporting Summary [file 41467_2023_38924_MOESM3_ESM.pdf]

nature portfolio

Corresponding author(s): Richard J. Lewis

Last updated by author(s): 18/05/2023

Reporting Summary

Nature Portfolio wishes to improve the reproducibility of the work that we publish. This form provides structure for consistency and transparency in reporting. For further information on Nature Portfolio policies, see our Editorial Policies and the Editorial Policy Checklist.

Statistics

For all statistical analyses, confirm that the following items are present in the figure legend, table legend, main text, or Methods section.

n/a	Confirmed	
☐	☒	The exact sample size (*n*) for each experimental group/condition, given as a discrete number and unit of measurement
☐	☒	A statement on whether measurements were taken from distinct samples or whether the same sample was measured repeatedly
☐	☒	The statistical test(s) used AND whether they are one- or two-sided<br>*Only common tests should be described solely by name; describe more complex techniques in the Methods section.*
☒	☐	A description of all covariates tested
☒	☐	A description of any assumptions or corrections, such as tests of normality and adjustment for multiple comparisons
☐	☒	A full description of the statistical parameters including central tendency (e.g. means) or other basic estimates (e.g. regression coefficient) AND variation (e.g. standard deviation) or associated estimates of uncertainty (e.g. confidence intervals)
☐	☒	For null hypothesis testing, the test statistic (e.g. *F*, *t*, *r*) with confidence intervals, effect sizes, degrees of freedom and *P* value noted<br>*Give P values as exact values whenever suitable.*
☒	☐	For Bayesian analysis, information on the choice of priors and Markov chain Monte Carlo settings
☒	☐	For hierarchical and complex designs, identification of the appropriate level for tests and full reporting of outcomes
☒	☐	Estimates of effect sizes (e.g. Cohen's *d*, Pearson's *r*), indicating how they were calculated

Our web collection on statistics for biologists contains articles on many of the points above.

Software and code

Policy information about availability of computer code

Data collection	Light microscopy (histological sections): Zeiss Zen Blue 3.2<br>Light microscopy (live animals): Leica LAS EZ 3.0.0<br>Transmission electron microscopy: Hitachi TEM System Model HT7700 02.30.15.56<br>Scanning electron microscopy: Hitachi TM4000 Tabletop Microscope 1.5<br>Slide scanner: Zeiss Zen 3.5<br>LC-MS and LC-MS/MS data: SCIEX OS 3.0.0.3339<br>MALDI-MS data: Bruker timsControl 3.1.4
Data analysis	Cropping and stacking of images for 3D volume generation: ImageJ/Fiji 2.0.0<br>Sections alignment, 3D volume generation and smoothing: Amira 2021.1<br>3D volume smoothing, image editing (brightness and contrast adjustment, background removal): Photoshop 21.2.1<br>Plotting of morphometric data from radular teeth: GraphPad Prism 9.4.1<br>Transcriptome de-novo assembly: Trinity 2.8.4<br>Transcriptomic analyses: BBTools 38.84<br>Classification of conotoxin transcripts into gene superfamilies and classes: ConoSorter 1.1<br>Search for N-terminal signal regions: SignalP 5.0<br>Computing of transcript expression levels in TPM: Kallisto 0.46.1<br>Principal component analysis: XLSTAT (free trial version) and GraphPad Prism 9.4.1<br>LC-MS data visualisation and analysis: SCIEX Peakview 2.2

Matching of CID-MS/MS data with RNA-seq database: ProteinPilot 5.0
Matching of EAD-MS/MS data with RNA-seq database: Mascot 2.5.1
MALDI-MS data visualisation and analysis: Bruker Data Analysis 6.0
Amino acid sequence alignment: MAFFT 7.504
Conotoxin gene evolutionary history: IQ-TREE 2.2

For manuscripts utilizing custom algorithms or software that are central to the research but not yet described in published literature, software must be made available to editors and reviewers. We strongly encourage code deposition in a community repository (e.g. GitHub). See the Nature Portfolio guidelines for submitting code & software for further information.

Data

Policy information about availability of data

All manuscripts must include a data availability statement. This statement should provide the following information, where applicable:

- Accession codes, unique identifiers, or web links for publicly available datasets
- A description of any restrictions on data availability
- For clinical datasets or third party data, please ensure that the statement adheres to our policy

All RNA sequencing data have been deposited in the NCBI Sequence Read Archive (SRA) under BioProject accession number PRJNA943605 [https://www.ncbi.nlm.nih.gov/sra/PRJNA943605]. Raw data for the adult C. magus from the Philippines are accessible under BioProject accession number PRJNA505200 [https://www.ncbi.nlm.nih.gov/bioproject/PRJNA505200]. Contigs were annotated using the non-redundant UniprotKB/SwissProt [http://www.uniprot.org] and Conoserver [https://www.conoserver.org/] databases. Conotoxin precursor sequences have been deposited in NCBI GenBank [https://www.ncbi.nlm.nih.gov/nuccore] under accession numbers OQ644315–OQ644445. Mass spectrometry proteomics data have been deposited in the ProteomeXchange Consortium via the PRIDE partner repository with the dataset identifier PXD042133 [http://www.ebi.ac.uk/pride/archive/projects/PXD042133]. Source data for radular tooth morphometry, principal component analysis and proteomics are available as Supplementary Data 1, 2 and 3, respectively.

Human research participants

Policy information about studies involving human research participants and Sex and Gender in Research.

Reporting on sex and gender	Not applicable
Population characteristics	Not applicable
Recruitment	Not applicable
Ethics oversight	Not applicable

Note that full information on the approval of the study protocol must also be provided in the manuscript.

Field-specific reporting

Please select the one below that is the best fit for your research. If you are not sure, read the appropriate sections before making your selection.

☐ Life sciences ☐ Behavioural & social sciences ☒ Ecological, evolutionary & environmental sciences

For a reference copy of the document with all sections, see nature.com/documents/nr-reporting-summary-flat.pdf

Ecological, evolutionary & environmental sciences study design

All studies must disclose on these points even when the disclosure is negative.

Study description	This study covers venom apparatus morphogenesis, feeding behaviour, radular tooth morphometry and venom composition during the development of Conus magus.
Research sample	The study was carried out on the cone snail Conus magus (Gastropoda, Conidae). One adult male and one adult female were sourced from the Great Barrier Reef, Queensland, Australia. Both specimens were kept in a 8 L breeding tank with larvae, metamorphic stages and juvenile specimens obtained from a single egg clutch.
Sampling strategy	No statistical method was used to predetermine sample size. For histology, sample size was determined to be adequate based on the consistency of results between samples and on similar observations by Page, 2012 on Conus lividus. For transmission electron microscopy of radular teeth, sample size was determined to be adequate based on the consistency of results between samples and on similar observations on wild-caught specimens by Nybakken & Perron, 1988. No sampling was performed for behavioural experiments and all observations were recorded. All remaining specimens were sacrificed for transcriptomics and proteomics studies.
Data collection	Feeding behavioural data were collected at night at the Institute for Molecular Bioscience (IMB), University of Queensland (UQ) by A.Rogalski (using pen and paper). Histological data were collected at IMB by A.Rogalski (on a computer). Images of live specimens and electron microscopy data (TEM and SEM) were collected at the Centre for Microscopy and Microanalysis (CMM, UQ) by A.Rogalski

	(on a computer). MALDI-MS data were collected at CMM by A.Rogalski (on a computer). LC-MS and LC-MS/MS data were collected at the School of Chemistry and Molecular Biosciences (SCMB, UQ) by Amanda Nouwens (on a computer). For transcriptomics, cDNA library preparation and sequencing were performed by the IMB Sequencing Facility, and raw data processing and de-novo assembly were performed by the Queensland Facility for Advanced Bioinformatics (UQ).
Timing and spatial scale	Data were collected between April 2020 and January 2023
Data exclusions	No data were excluded from the analyses except for one RNA-seq sample (20 juvenile venom glands pooled) with poor yield insufficient for subsequent sequencing.
Reproducibility	While this study presents data collected from a single egg-laying event, the culture of C. magus from eggs through metamorphosis was successfully repeated three times during the year 2020, yielding similar behavioural observations. The robustness of the rearing protocol was validated by the successful culture of two other cone snail species (Rogalski, A. et al., manuscript in preparation).
Randomization	All collected samples were analysed and there was no need for randomisation.
Blinding	Blinding was not relevant to this study because no bias could be made by the subject or the tester in the experiments performed.

Did the study involve field work? ☐ Yes ☒ No

Reporting for specific materials, systems and methods

We require information from authors about some types of materials, experimental systems and methods used in many studies. Here, indicate whether each material, system or method listed is relevant to your study. If you are not sure if a list item applies to your research, read the appropriate section before selecting a response.

Materials & experimental systems

n/a	Involved in the study
☒	☐ Antibodies
☒	☐ Eukaryotic cell lines
☒	☐ Palaeontology and archaeology
☐	☒ Animals and other organisms
☒	☐ Clinical data
☒	☐ Dual use research of concern

Methods

n/a	Involved in the study
☒	☐ ChIP-seq
☒	☐ Flow cytometry
☒	☐ MRI-based neuroimaging

Animals and other research organisms

Policy information about studies involving animals; ARRIVE guidelines recommended for reporting animal research, and Sex and Gender in Research

Laboratory animals	Two adult specimens of Conus magus (one male and one female) were purchased from Cairns Marine (Cairns, QLD, Australia) and kept together in our in-house marine system as described in Methods. At the end of the experiments, the female specimen was sacrificed and the venom gland dissected for histology, transcriptomics and proteomics analyses. Larvae, metamorphic stages and juvenile specimens were all obtained from a single egg clutch laid in our marine system.
Wild animals	No wild animals were used in this study.
Reporting on sex	As stated above, the study includes two adult specimens of Conus magus, one male and one female.
Field-collected samples	No field-collected samples were used in this study.
Ethics oversight	Use of zebrafish (Danio rerio) for cone snail feeding was approved by the University of Queensland Animal Ethics Committee (AEC No. 2019/AE000271).

Note that full information on the approval of the study protocol must also be provided in the manuscript.
