# Supplementary material for: Coordinated adaptations define the ontogenetic shift from worm- to fish-hunting in a venomous cone snail
Source: Nat Commun. 2023 Jun 13;14:3287. doi: 10.1038/s41467-023-38924-5 (PMC10264353; doi:10.1038/s41467-023-38924-5)
Supplement: Supplementary file 4 — Description of Additional Supplementary Files [file 41467_2023_38924_MOESM4_ESM.docx]

**Description of Additional Supplementary Files**

Supplementary Data 1

Description: Radular tooth morphometry in juvenile C. magus and adult worm-, mollusc- and fishhunters. Data for adult radular teeth are from Mosquera and Massilia, 2002.

Supplementary Data 2

Description: Filtered conotoxin precursor sequences from our RNAseq experiments (sheet 1) and C. magus venom database including previously reported sequences (sheet 2). Sequences lacking stop codons were not included.

Supplementary Data 3

Description: Lists of masses from LCMS and MALDI-MS experiments.

Supplementary Data 4

Description: Principal component analysis data matrix, summary statistics, contribution of variables, score and loading plots.

Supplementary Movie 1

Description: Female C. magus depositing an egg capsule under a coral rock.

Supplementary Movie 2

Description: Juvenile C. magus feeding on a polychaete worm
